# Triple burden of malnutrition among Malaysian children aged 6 months to 12 years: current findings from SEANUTS II Malaysia

**DOI:** 10.1017/S1368980023002239

**Published:** 2023-11-07

**Authors:** Bee Koon Poh, Jyh Eiin Wong, Shoo Thien Lee, Jasmine Siew Min Chia, Giin Shang Yeo, Razinah Sharif, Nik Shanita Safii, Nor Aini Jamil, Caryn Mei Hsien Chan, Nor MF Farah, Mohd Jamil Sameeha, Denise Koh, Nur Zakiah Mohd Saat, See Meng Lim, A Karim Norimah, Abd Talib Ruzita, Siti Balkis Budin, Lei Hum Wee, Swee Fong Tang, Ilse Khouw

**Affiliations:** 1 Faculty of Health Sciences, Universiti Kebangsaan Malaysia, 50300 Kuala Lumpur, Malaysia; 2 Faculty of Health & Life Sciences, Management & Science University, 40100 Shah Alam, Selangor, Malaysia; 3 School of Pharmacy, Management & Science University, 40100 Shah Alam, Selangor, Malaysia; 4 Faculty of Education, Universiti Kebangsaan Malaysia, 43600 Bangi, Selangor, Malaysia; 5 School of Medicine, Taylor’s University, 47500 Subang Jaya, Selangor, Malaysia; 6 Specialist Children’s Hospital, Universiti Kebangsaan Malaysia, 56000 Kuala Lumpur, Malaysia; 7 FrieslandCampina, Amersfoort, The Netherlands

**Keywords:** Children, dietary intake, malnutrition, nutritional biomarkers, nutritional status

## Abstract

**Objective:**

This paper aims to report South East Asian Nutrition Surveys (SEANUTS) II Malaysia data on nutritional status, dietary intake and nutritional biomarkers of children aged 6 months to 12 years.

**Design:**

Cross-sectional survey conducted in 2019–2020.

**Setting:**

Multistage cluster sampling conducted in Central, Northern, Southern and East Coast regions of Peninsular Malaysia.

**Participants:**

2989 children aged 0·5–12·9 years.

**Results:**

Prevalences of stunting, thinness, overweight and obesity among children aged 0·5–12·9 years were 8·9 %, 6·7 %, 9·2 % and 8·8 %, respectively. Among children below 5 years old, 11·4 % were underweight, 13·8 % had stunting and 6·2 % had wasting. Data on nutritional biomarkers showed that a small proportion of children aged 4–12 years had Fe (2·9 %) and vitamin A deficiencies (3·1 %). Prevalence of anaemia was distinctly different between children below 4 years old (40·3 %) and those aged 4 years and above (3·0 %). One-fourth of children (25·1 %) had vitamin D insufficiency, which was twice as prevalent in girls (35·2 % *v*. boys: 15·6 %). The majority of children did not meet the recommended dietary intake for Ca (79·4 %) and vitamin D (94·8 %).

**Conclusions:**

Data from SEANUTS II Malaysia confirmed that triple burden of malnutrition coexist among children in Peninsular Malaysia, with higher prevalence of overnutrition than undernutrition. Anaemia is highly prevalent among children below 4 years old, while vitamin D insufficiency is more prevalent among girls. Low intakes of dietary Ca and vitamin D are also of concern. These findings provide policymakers with useful and evidence-based data to formulate strategies that address the nutritional issues of Malaysian children.

The double burden of malnutrition, characterised as the simultaneous manifestation of both undernutrition and overweight/obesity, has become a major public health issue and is particularly prominent in low- to middle-income countries. Nine of the eleven countries in Southeast Asia have moderate to very high prevalences of stunting (≥ 20 %) and wasting (≥ 5 %)^(1)^. The prevalence of children under 5 years old with either overweight or micronutrient deficiencies is approximately 50 % in Southeast Asia^(1)^. The coexistence of underweight, overweight and micronutrient deficiencies, commonly referred to as the triple burden of malnutrition, must be addressed to ensure that the long-term effects, especially in children, are halted early. This is in line with the global call for action through Goal 2 of the UN Sustainable Development Goals to eliminate hunger and malnutrition by the year 2030^(2)^.

South East Asian Nutrition Surveys (SEANUTS) is a multi-country collaborative survey to assess the current nutritional status and dietary patterns of children aged 6 months to 12 years. SEANUTS I was conducted between 2010 and 2011 simultaneously in four countries, namely Malaysia, Indonesia, Thailand and Vietnam^(3)^. SEANUTS I highlighted the issues of malnutrition in Southeast Asia, where overweight and obesity rates were more prevalent in urban areas, while children in rural areas were leaning towards undernutrition^(4–7)^. In Malaysia, the prevalences of overweight (9·8 %) and obesity (11·8 %) were higher than the prevalence of thinness (5·4 %) and stunting (8·4 %), and these trends were observed across all age groups^(7)^.

SEANUTS I findings have impacted national planning. In Malaysia, for example, actions were initiated following reports of the double burden of malnutrition, as well as a high prevalence of vitamin D insufficiency^(7)^. The evidence gathered were also used to establish the targets for the National Plan of Action for Nutrition of Malaysia (NPANM) 2016–2025^(8)^, which was developed in line with the WHO’s Global Nutrition Targets 2025 to reduce the prevalence of stunting in children under 5 years, to reduce and maintain childhood wasting below 5 % and to ensure no increase in childhood overweight^(9)^.

Unfortunately, there has not been much progress in the improvement of the nutritional status of Malaysian children aged 6 months to 12·9 years, since the findings of SEANUTS I Malaysia were released. Subsequently, the National Health and Morbidity Survey (NHMS), a nationally representative Malaysian health survey, reported the nutritional status of children below 5 years old, with increasing trends in both underweight [11·6 % (2011); 12·4 % (2015); 14·1 % (2019)] and stunting [16·6 % (2011); 17·7 % (2015); 21·8 % (2019)], although childhood wasting showed a gradual decline [12·4 % (2011); 8·1 % (2015); 9·7 % (2019)]^(10–12)^. Furthermore, the prevalence of obesity among children below 18 years had increased, whereby it doubled from 6·1 % in 2011 to 11·9 % in 2015 and then jumped to 14·8 % in 2019^(10–12)^.

Apart from SEANUTS I Malaysia, there has not been any nationwide study to date, including the nationally representative NHMS, which provides comprehensive data on nutritional status including anthropometric measurements, nutritional biomarkers, dietary intakes and other nutrition-related parameters among Malaysian children. The second SEANUTS study (SEANUTS II), which was conducted about a decade after SEANUTS I, aims to continue providing current and overarching information regarding the nutritional status of children across Southeast Asia. Therefore, this paper aims to present an up-to-date overview of the nutritional status, dietary intake and nutritional biomarkers of children aged 6 months to 12 years in Peninsular Malaysia.

## Methodology

### Study design and scope

SEANUTS II Malaysia was designed as a cross-sectional study to obtain comprehensive nutrition information of children aged 0·5 to 12·9 years from six regions of Malaysia, namely Central, East Coast, Northern, and Southern regions of Peninsular Malaysia, as well as Sabah and Sarawak. Data collection, employing multistage cluster sampling approach, was conducted from May 2019 to March 2020.

The first stage of sampling was the selection of districts. In each region, two districts (one urban and one rural) from different states were randomly selected by the Department of Statistics of Malaysia (DOSM) to represent the respective regions. The selected urban areas are gazetted areas with their adjoining built-up areas that have a combined population of 10 000 and above^(13)^, including: Kuala Lumpur Federal Territory (Central region); Kuantan, Pahang (East Coast region); the Northeast district of Penang Island (Northern region); Seremban, Negeri Sembilan (Southern region); Kota Kinabalu (Sabah); and Kuching (Sarawak). As for the rural areas, gazetted or non-gazetted areas with population less than 10 000^(13)^ were selected: the districts of Kuala Langat, Selangor (Central region); Pasir Mas, Kelantan (East Coast region); Kerian, Perak (Northern region); Kota Tinggi, Johor (Southern region); Kinabatangan (Sabah); and Serian (Sarawak).

The second stage was sampling within the selected districts. In each district, DOSM provided a list of randomly selected enumeration blocks for the sampling of home-based participants. Subjects were recruited from all districts within a 5 km radius from each selected enumeration block, using either home-based or school-based approaches. A home-based approach was conducted for children aged 0·5 to 6·9 years who were not attending primary schools. The children were recruited primarily through home visits. For young children who were attending preschools, data collection was conducted at their nurseries or kindergartens. Children aged 7·0 to 12·9 years were recruited from primary schools within the selected areas.

### Sample size estimation

To ensure that the sample size (based on prevalence) was adequate, the following formula was used^(14)^:

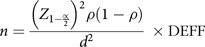




The power analysis for estimating the number of children (*n*) was based on the six regions of study location. Obesity prevalence ( 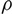 ) of Malaysian children was taken from SEANUTS I Malaysia^(7)^. The obesity prevalence was 11·8 %^(7)^, 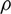 was set to 0·12. 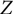 was the critical value of 1·96 for the corresponding 95 % confidence level ( 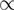 : 0·05), while 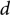 was the tolerable error of 6 %. The calculated number was 113 and was adjusted for design effect (DEFF) of 2, resulting in a sample of 225 for each region. In anticipation of a 70 % completion rate (based on unpublished results from SEANUTS I), the final sample for each region was calculated (225 × 100/70) and the final sample size for each region was determined to be 322. For the six regions, a total of (322 × 6) or 1932 children were required for each recruitment approach (i.e. the home- and school-based approaches). Therefore, the final required sample for this study was doubled (1932 × 2) to 3864.

A subsample of children aged 4 years and above was recruited for nutritional biomarkers analysis. According to SEANUTS I Malaysia, of all the nutritional biomarkers, the prevalence of vitamin D deficiency was the highest^(7)^. Hence, this prevalence was used to calculate the size of the SEANUTS II Malaysia subsample for the nutritional biomarkers study. Using the same formula as above, based on the prevalence of vitamin D deficiency, 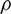 was 0·04^(7)^, 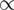 was 0·05, z was 1·96, 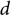 was 9 % and design effect was 2. The sample of each region was 36. In anticipation of a 50 % response rate, the final sample of each region was determined to be (36 × 100/50) or 72. A total of 432 children were required for each of the two recruitment approaches. Thus, a final subsample of 864 was needed for the nutritional biomarker study.

**Fig. 1:**
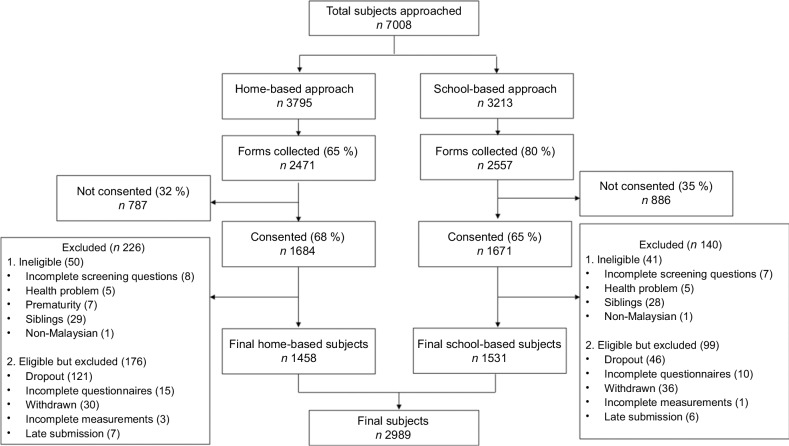
Flow diagram of subject recruitment

In early 2020, SEANUTS II Malaysia was in the midst of data collection when the COVID-19 pandemic began. Recruitment of subjects was halted. Data collection of subjects from two regions in East Malaysia, namely Sabah and Sarawak, was not yet conducted at that point in time. Data collection from children in four regions of Peninsular Malaysia, namely the Central, Northern, Southern and East Coast regions, had been completed just a couple of weeks prior to the announcement on 16 March 2020 of the COVID-19 pandemic movement control order in Malaysia. Hence, the final pool of subjects in this study consisted only of children aged 6 months to 12 years in Peninsular Malaysia. Figure 1 shows the flow diagram of subject recruitment, including response rate, subject exclusion and the final number of valid subjects for data analysis.

### Subjects

Children must be of Malaysian nationality and aged between 6 months and 12 years to be included in this study. The number of boys and girls recruited were in accordance with the national gender ratio for their area of residence (urban/rural). Children who were unwell on the measurement day, had a physical disability, or presented with medical history or illness that could have impacted their usual diet and physical activity were excluded from the study. If there were two or more siblings who fulfilled the study inclusion criteria, only one of the siblings was recruited to participate in the study. Based on the age of the subjects at the time of measurement, they were then categorised as either infants (0·5–0·9 years), toddlers (1·0–3·9 years), preschoolers (4·0–6·9 years) or school-aged children (7·0–12·9 years).

### Ethics approval and permission for data collection

The protocols and materials of the present study were reviewed and approved by the Universiti Kebangsaan Malaysia Research Ethics Committee. Written informed consent was obtained from a parent or guardian prior to a child’s participation in the study, and verbal assent was obtained from each child before data collection. This study was conducted according to the principles set forth in the Declaration of Helsinki. It is registered in the Dutch Trial Registry (Reference: NL7975). Permission to conduct data collection was obtained from all relevant parties, including the Malaysian Ministry of Education, Departments of Education at the state level, the Department of Community Development (KEMAS) under the Ministry of Rural Development, the Department of National Unity and Integration under the Ministry of National Unity, school principals, kindergartens, and nurseries, as well as community leaders.

### Data collection procedure

A detailed description of the study protocol is described in the SEANUTS II study design paper^(15)^. Prior to the commencement of data collection, all research team members and enumerators underwent comprehensive training on all assessment methods and study procedures. Data collection of all measurements were scheduled after a parent or guardian gave consent and completed the sociodemographic questionnaire. Prior to the day of data collection, parents or guardians were reminded and asked to ensure their children were dressed in sports attire on the day of measurement. On the day of data collection, the children were assigned to stations for anthropometric measurement and dietary recall. Child anthropometric measurements were measured by trained researchers. Dietary recall interviews were conducted with parents/caregivers for children below 10 years old and directly with children aged 10 to 12 years. When necessary, telephone calls to a parent or guardian were made to verify and complete the questionnaire, as well as to conduct dietary recall interviews for children aged below 10 years. Blood withdrawal was conducted among a subsample on a separate day. Subjects who completed the physical measurements and questionnaires were given a health report, a certificate of participation and a small token of appreciation.

### Anthropometric measurements

All anthropometric measurements were done using standardised procedures by trained researchers. Bi-yearly technical error of measurement for anthropometric assessment was also conducted to ensure intra- and inter-observer variations were minimal among researchers. Measurements were taken in duplicates, and the average value was used as the final value. In cases where deviation between the two readings was higher than the maximum allowable difference, a third measurement was taken, and the median was used as the final value. Children were asked to dress in light clothing, with shoes not worn during measurement.

Body weight was measured using SECA 354 digital weighing scale (seca GmbH) to the nearest 0·005 kg for infants, or SECA 874 (seca GmbH) to the nearest 0·05 kg for older children. Recumbent length for children aged below 2 years was measured using SECA 210 measuring mat (seca GmbH) or SECA 417 infantometer (seca GmbH) to the nearest 0·1 cm. Standing height of older children was measured using SECA 213 stadiometer (seca GmbH) to the nearest 0·1 cm. The maximum allowable difference was set at less than 0·1 kg for body weight and less than 0·5 cm for height or recumbent length. BMI was calculated by dividing the measured weight (in kg) by the square of height (in metre).

The anthropometric status of subjects was classified using the WHO child growth standards for 0–5 years^(16)^ and the WHO growth reference for 5–19 years^(17)^. Z-scores for weight-for-age (WAZ), height-for-age (HAZ), BMI-for-age (BAZ) and weight-for-height (WHZ) were determined using WHO Anthro version 3.2.2 (WHO) software for children aged below 5 years^(18)^. For children aged 5 years and above, the WHO AnthroPlus version 1.0.4 (WHO) software was used to determine the WAZ, HAZ and BAZ^(19)^. The cut-off values for wasting, stunting and thinness for all children are -2 sd. The cut-off values for overweight and obesity among children aged below 5 years are +2 sd and +3 sd, respectively, whereas the cut-off among children aged 5 years and above are +1 sd and +2 sd, respectively, as defined by the WHO^(16,17)^. Sixteen children with implausible Z-score values were excluded, when WAZ < -5 sd or WAZ > 5 sd, HAZ < -6 sd or HAZ > 6 sd, WHZ < -6 sd or WHZ > 5 sd, or BAZ < -5 sd or BAZ > 5 sd ^(19,20)^.

### Assessment of dietary intake

Dietary intake was assessed using 1-d triple-pass 24-h dietary recall interview from 12 am to 12 am the following day^(21)^. Parents, guardians or caregivers were encouraged to capture images of all food consumed by the children on the day prior to the dietary recall interview. Additionally, a probing guide was used to help recollect foods that might typically be overlooked or forgotten during the interview. In cases where children encountered difficulties recalling, any uncertainties were resolved through seeking clarifications from parents/guardians/caregivers. Household measures were used as portion size estimation aid during the interviews. For subjects aged 6 months to 9 years, dietary recall was proxy-reported by either parents, guardians or caregivers through face-to-face or telephone interview (with a soft copy of the household measurement booklet for reference). Dietary intake for children aged 10 to 12 years old was self-reported through face-to-face interviews conducted during school visit days. Nutrient analysis was conducted using Nutritionist Pro software (Axxya Systems), with nutrient values obtained mainly from the Malaysian Food Composition Database^(22)^, and supported by USDA^(23)^, UK^(24)^, FOCOS^(25)^, and food product labels.

Energy and nutrient intake values were compared with estimated average requirement (EAR) and recommended nutrient intake (RNI) 2017 for Malaysia^(26)^. EAR was derived from the Malaysian RNI by subtracting twice the CV provided by the Institute of Medicine (IOM)^(27–30)^. If RNI and EAR were unavailable, adequate intake as reported in the Malaysian RNI^(26)^ was used. The EAR for Fe was calculated from the total absolute requirement at the median level provided by the WHO/FAO (2004)^(31)^ as shown below:

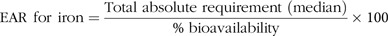




where Total absolute requirement = Requirement for growth, basal losses + menstrual losses^(31)^, % bioavailability =15 %

Prior to dietary data analysis, the ratio of reported energy intake (EI) and predicted energy expenditure (EE) was calculated based on the Black and Cole formula^(32)^ to exclude implausible data reporting. EE was estimated on the multiplication of BMR^(33–35)^ and physical activity level (PAL)^(36)^. For PAL value in the formula, children were assumed to have a moderate level of physical activity (children aged 0·5–5·9 years: PAL 1·60; boys aged 6·0–12·9 years: PAL 1·75; girls aged 6·0–12·9 years: PAL 1·70)^(36)^. According to the formula proposed by Black and Cole^(32)^, 99 % CI, 8·2 % of within-subject variation in EE and 23 % of within-subject variation in EI were applied in the present study^(37)^:

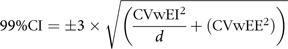




where CV wEE = within-subject variation in of EE, 8·2 %, CV wEI = within-subject variation in EI, 23 %, and *d* = number of days of diet assessment.

The final acceptable reporting range was 0·27–1·73, and only subjects with EI:EE ratio within this range were included for further dietary analysis. A total of 108 children with under- or over-reporting were excluded. Nutrient analysis did not include intake of dietary supplements.

### Sociodemographic questionnaire

A questionnaire was used to collect data regarding sociodemographic background. All questions were self-administered by parents or guardians in Malay–English or Mandarin–English language versions. The sociodemographic questionnaire (SES) comprised thirty-three items requesting both child and parents’ or guardians’ information, as well as total household monthly income information.

### Analysis of nutritional biomarkers

The nutritional biomarkers investigated in SEANUTS II Malaysia included full blood count, Hb, Fe, ferritin, transferrin saturation, alpha-1-acid glycoprotein (AGP), C-reactive protein (CRP), vitamin B_12_, vitamin A, vitamin D, and fasting blood glucose levels, as well as lipid profile and metabolomics, as described by Tan et al.^(15)^, though this paper will focus only selected parameters. In a subsample of children aged 0·5 to 3·9 years, Hb was measured using the finger-prick method following standardised protocols. Approximately 10 μL of capillary blood was collected in the microcuvette for measurement by HemoCueHb201+ system (HemoCue AB).

Venous blood was drawn by trained phlebotomists from a subsample of children aged 4 years and above for nutritional biomarkers analysis. The children who were involved in nutritional biomarkers analysis were required to fast overnight for 8–10 h before blood withdrawal the following morning. Approximately 13 mL of venous blood were drawn and aliquoted into BD Vacutainer^®^ SST^TM^, EDTA and fluoride tubes. The collected blood samples were kept at 4°C in a standard storage box with ice packs and transported immediately to an accredited lab. Standard methods of lab analysis, such as flow cytometry (Hb), spectrophotometry (CRP and ferritin), ECLIA [vitamin B_12_ and vitamin D (25(OH)D)] and liquid–liquid extraction (vitamin A), were conducted. Serum AGP was measured according to the manufacturer’s instructions using commercial ELISA kit (Immunology Consultants Laboratory, Inc.).

Anaemia was defined as Hb level < 110 g/L for children aged < 5 years, < 115 g/L for children aged 5–11·9 years and < 120 g/L for children aged ≥ 12 years^(38)^. Fe deficiency was defined as ferritin level < 12 µg/L for children < 5 years and < 15 µg/L for children ≥ 5 years^(39)^. Ferritin level was adjusted by multiplying a correction factor of 0·77 for incubation stage (CRP > 5 mg/L; AGP ≤ 1 g/L), 0·53 for early convalescence stage (CRP > 5 mg/L; AGP > 1 g/L) and 0·75 for late convalescence stage (CRP ≤ 5 mg/L; AGP > 1 g/L)^(40)^. Serum retinol level 0·35–0·70 µmol/L was defined as mild vitamin A deficiency and < 0·35 µmol/L as severe vitamin A deficiency^(41)^. Serum 25(OH)D < 50 nmol/L and < 25 nmol/L were defined as vitamin D insufficiency and deficiency^(42)^, respectively, while vitamin B_12_ level < 150 pmol/L was defined as vitamin B_12_ deficiency^(43)^.

### Data management and statistical analysis

Data were transferred from paper forms and questionnaires into an online electronic data capture system (Viedoc Technologies). The quality of data collected was checked in two steps: first, by data checking all entries in Viedoc against paper forms and questionnaires and then rechecking 20 % of the Viedoc entries against paper forms to validate the first-round data entry.

Statistical analyses were performed using IBM SPSS Statistics for Windows version 22.0 (IBM Corp.), employing the complex sampling module. Weight factor was calculated based on the projected Malaysian population aged 6 months to 12 years in 2019, using the Malaysian census database in 2010^(44)^. Descriptive analysis was performed and presented as mean and standard error. ANCOVA after adjusting for age was used to examine the mean difference of anthropometric measurements, dietary intake, and nutritional biomarkers between sexes and between areas of residence. Nutritional status, nutritional biomarkers deficiency and children not achieving dietary recommendations were reported in percentages. Pearson’s Chi-square was performed to examine the percentage difference in nutritional status, nutritional biomarkers deficiency, and achievement of dietary intake recommendations between boys and girls, as well as between urban and rural children. Significance was determined using two-sided tests, where *p*-value less than 0·05 indicated statistical significance.

## Results

A total of 2989 children, representing an estimated 4 936 600 Malaysian children aged 0·5 to 12·9 years in Peninsular Malaysia (Table 1), were studied. The sociodemographic characteristics of children by recruitment approaches is reported in Supplementary Table 1. The nutritional status of children is reported in Table 2. The prevalences of stunting, overweight and obesity for children aged 0·5 to 12·9 years were 8·9 %, 9·2 % and 8·8 %, respectively. There was no significant difference in stunting, underweight, wasting and thinness between the sexes and area of residence, with the exception of older girls aged 7·0–12·9 years living in rural areas, who had significantly higher prevalence of stunting compared to their male counterparts (11·3 % *v*. 2·9 %). Meanwhile, the prevalences of overweight (15·0 %) and obesity (14·7 %) were highest in older children (7·0–12·9 years old). Comparison between the sexes showed that the overall prevalence of obesity was significantly higher among boys, and this trend is consistent for the 7·0–12·9 age group. The prevalences of stunting, underweight, wasting and thinness for children below 5 years old were 13·8 %, 11·4 %, 6·2 % and 5·8 %, respectively (Supplementary Table 2). The anthropometric characteristics of children for each age group are reported in Supplementary Table 3.

**Table 1.** Distribution of subjects by age group, area of residence and sex

	0·5–0·9 years		1·0–3·9 years		4·0–6·9 years		7·0–12·9 years		0·5–12·9 years	
	Sample (n)	Estimated population	Sample (n)	Estimated population	Sample (n)	Estimated population	Sample (n)	Estimated population	Sample (n)	Estimated population
All										
Boys	39	104 950	274	604 400	468	625 100	651	1 217 400	1432	2 551 850
Girls	39	89 550	284	577 200	458	587 600	776	1 130 400	1557	2 384 750
All	78	194 500	558	1 181 600	926	1 212 700	1427	2 347 800	2989	4 936 600
Urban										
Boys	27	74 394	176	410 170	343	485 798	459	940 460	1005	1 910 822
Girls	24	59 678	207	417 522	335	452 310	532	780 067	1098	1 709 577
All	51	134 072	383	827 692	678	938 108	991	1 720 527	2103	3 620 399
Rural										
Boys	12	30 556	98	194 230	125	139 302	192	276 940	427	641 028
Girls	15	29 872	77	159 678	123	135 290	244	350 333	459	675 173
All	27	60 428	175	353 908	248	274 592	436	627 273	886	1 316 201

Projection population 2020 based on census 2010 (DOSM 2010)^(44)^.

**Table 2.** Percentage of stunted, wasted, underweight, thin, overweight and obese children per age group

	0·5–0·9 years			1·0–3·9 years			4·0–6·9 years			7·0–12·9 years			0·5–12·9 years		
	Boys	Girls	All	Boys	Girls	All	Boys	Girls	All	Boys	Girls	All	Boys	Girls	All
All															
Stunted	21·1	10·1	16·0	14·6	14·2	14·4	9·0	8·8	8·9	4·2	7·1	5·6	8·5	9·3	8·9
Underweightα	28·9	14·2	22·0	11·2	9·8	10·5	5·2	12·5	8·8	–	–	–	11·8	10·9	11·4
Wastedα	10·7	5·3	8·2	5·5	5·8	5·6	4·8	9·1	6·9	–	–	–	5·9	6·5	6·2
Wasting/thinness	10·4	3·1	7·0	5·6	4·7	5·2	6·2	5·9	6·1	6·9	8·8	7·8	6·6	6·9	6·7
Overweight	1·3	0	0·7	3·1	2·1	2·6	5·8	5·7	5·7	14·8	15·1	15·0	9·3	9·1	9·2
Obese	0	0	0	0·5	0·5	0·5	7·3	5·9	6·6	18·2**	11·0	14·7	10·6**	6·8	8·8
Urban															
Stunted	23·7	9·0	17·0	13·9	13·7	13·8	8·4	8·1	8·3	4·6	5·2†	4·9	8·3	8·2	8·2
Underweightα	36·0	17·9	27·8	9·2	8·5	8·9	4·9	13·8	9·3	–	–	–	11·1	10·7	10·9
Wastedα	10·1	3·7	7·2	4·2	5·2	4·7	5·3	9·6	7·4	–	–	–	5·1	6·1	5·6
Wasting/thinness	11·0	3·7	7·7	3·8	4·0	3·9	6·8	6·7	6·8	6·6	8·3	7·4	6·2	6·7	6·4
Overweight	1·9	0	1·0	3·9	2·0	2·9	6·1	6·2	6·2	15·4	14·5	15·0	10·1	8·8	9·5
Obese	0	0	0	0	0	0	7·0	5·9	6·4	16·8**	10·0	13·7	10·1**	6·1	8·2
Rural															
Stunted	15·0	12·5	13·8	16·0	15·5	15·7	10·9	10·9	10·9	2·9**	11·3	7·7	9·3	12·2	10·8
Underweightα	12·0	6·8	9·5	15·3	13·1	14·3	6·5	7·5	7·0	–	–	–	13·4	11·3	12·4
Wastedα	12·0	8·6	10·3	8·2	7·6	7·9	3·0	7·5	5·1	–	–	–	7·7	7·7	7·7
Wasting/thinness	9·0	1·8	5·4	9·4	6·5	8·1	4·4	3·0	3·7	8·0	10·0	9·1	7·7	7·4	7·5
Overweight	0	0	0	1·3	2·2	1·7	4·7	3·9	4·3	12·7	16·5	14·9	6·9	9·9	8·4
Obese	0	0	0	1·5	1·7	1·6	8·4	6·0	7·2	23·2*	13·4	17·6	12·2	8·6	10·3

α The data analyses involved children below 5 years old only.

Percentage values were significantly different from girls of each age group based on complex sampling Pearson’s Chi-square: *p < 0.05, **p < 0.01 and ***p < 0.001.

Percentage values were significantly different from rural children based on complex sampling Pearson’s Chi-square: †*p* < 0.05, ††*p* < 0.01 and †††*p* < 0.001.

Definition of nutritional status: stunted: height-for-age (HAZ) <-2 sd from the median; underweight (under 5 years only): weight-for-age (WAZ) <-2 sd from the median; wasted (under 5 years only): weight-for-height (WHZ) <-2 sd from the median; wasting (under 5 years): BMI-for-age (BAZ) <-2 sd from the median; thinness (5–12 years only): BMI-for-age (BAZ) <-2 sd from the median; overweight: BMI-for-age (BAZ) >2 sd (< 5 years) and >1 sd (5–12 years) from the median; obese: BMI-for-age (BAZ) >3 sd (< 5 years) and >2 sd (5–12 years) from the median.

Table 3 shows the nutritional biomarkers averages for children aged 4 years and above, while Supplementary Table 4 provides the Hb levels of children below 4 years. Table 4 reports the prevalences of anaemia, Fe deficiency, vitamin A deficiency and vitamin D insufficiency. The overall prevalence of anaemia among children aged 4·0 to 12·9 years was 3·0 %, with a higher prevalence in boys, compared to girls (4·7 % *v*. 1·2 %). A high prevalence of anaemia, about 40·3 %, was observed among children below 4 years old. The prevalences of Fe deficiency and vitamin A deficiency among children aged 4·0 to 12·9 years were 2·9 % and 3·1 %, respectively. Girls in the younger age group (4·0–6·9 years) exhibited higher Fe and vitamin A deficiencies, compared to boys. Comparison by area of residence shows that prevalence of vitamin A deficiency was higher among children in the rural areas (6·9 % *v*. 1·8 %). Three girls (0·3 %) had Fe deficiency anaemia, and one girl had severe vitamin A deficiency (data not shown). A quarter of children (25·1 %) aged 4·0 to 12·9 years had vitamin D insufficiency, with only three children having vitamin D deficiency (data not shown). Girls had higher vitamin D insufficiency than their male counterparts (35·2 % *v*. 15·6 %). None of the children in this study had vitamin B_12_ deficiency.

**Table 3.** Nutritional biomarkers of children by age groups, sex and area of residences

	4·0–6·9 years						7·0–12·9 years						4·0–12·9 years					
	Boys		Girls		All		Boys		Girls		All		Boys		Girls		All	
	Mean	se	Mean	se	Mean	se	Mean	se	Mean	se	Mean	se	Mean	se	Mean	se	Mean	se
All																		
Hb (g/L)	128·8	1·0	127·4	0·9	128·1	0·7	131·9	1·0	133·0	0·7	132·5	0·6	130·8	0·8	131·1	0·6	131·0	0·5
Ferritin (ug/L)	55·0	4·0	55·2	3·8	55·1	2·9	53·6	3·4	53·9	2·2	53·8	2·0	54·1	2·6	54·4	2·0	54·2	1·6
VitaminA/retinol (μmol/L)	1·14	0·04	1·09	0·03	1·12	0·03	1·12	0·03	1·14	0·02	1·13	0·02	1·13	0·02	1·13	0·02	1·13	0·01
Vitamin D (nmol/L)	79·0	2·8	72·6	2·7	75·8	2·2	63·2***	1·5	54·6	1·5	58·9	1·1	68·7***	1·4	60·8	1·4	64·7	1·0
Vitamin B_12_ (pmol/L)	691·7	27·4	763·3	34·8	727·5	22·2	550·3	14·3	574·0	19·5	562·2	12·1	598·9	13·2	639·2	17·7	619·1	11·0
Urban																		
Hb (g/L)	128·0	1·1	127·0	1·0	127·5†	0·8	132·1	1·1	132·3	0·8	132·2	0·7	130·7	0·8	130·5	0·7	130·6	0·6
Ferritin (ug/L)	55·8	4·7	54·8	4·4	55·3	3·2	53·0	3·6	56·8	2·8	54·7	2·7	54·1	2·8	56·1	2·4	55·0	1·9
Vitamin A/ retinol (μmol/L)	1·17	0·04	1·10	0·04	1·14	0·03	1·16†	0·03	1·17	0·03	1·16†	0·02	1·16††	0·03	1·14	0·02	1·15††	0·02
Vitamin D (nmol/L)	77·2	3·1	71·2	3·1	74·2†	2·2	61·2**†	1·7	53·7	1·9	57·8	1·3	66·7**††	1·6	59·6	1·7	63·4††	1·2
Vitamin B_12_ (pmol/L)	720·7††	30·8	771·5	40·6	745·7†	25·5	545·1	16·5	532·0††	24·9	539·2††	14·4	605·7	15·3	619·4	22·4	612·2	13·1
Rural																		
Hb (g/L)	132·2	2·0	129·2	2·0	130·8	1·5	131·5	2·2	134·1	1·3	133·0	1·2	131·4	1·8	132·5	1·1	132·0	1·0
Ferritin (ug/L)	51·5	6·4	56·7	5·1	53·9	4·3	55·4	8·9	49·0	3·5	51·6	4·2	54·0	6·5	50·5	3·2	52·1	3·4
VitaminA/ retinol (μmol/L)	1·02	0·07	1·04	0·04	1·03	0·04	1·02	0·06	1·11	0·04	1·07	0·03	1·02	0·05	1·09	0·03	1·06	0·03
Vitamin D (nmol/L)	86·4	5·8	79·2	3·9	83·2	3·6	69·3***	2·7	56·2	2·5	61·6	1·9	75·4***	2·6	63·6	2·2	68·9	1·8
Vitamin B_12_ (pmol/L)	577·8*	43·2	724·6	51·9	643·3	35·8	566·1*	27·2	646·4	28·7	613·2	20·8	576·4**	26·6	685·3	26·1	636·2	19·4

Mean values were significantly different from girls of each age group based on complex sampling ANCOVA after adjusted for age: *p < 0·05, **p < 0·01 and ***p < 0·001.

Mean values were significantly different from rural children based on complex sampling ANCOVA after adjusted for age: †*p* < 0·05, ††*p* < 0·01 and †††*p* < 0·001.

**Table 4.** Prevalences of anaemia, Fe deficiency, vitamin A deficiency and vitamin D insufficiency by age group

	0·5–3·9 years			4·0–6·9 years			7·0–12·9 years			4·0–12·9 years		
	Boys	Girls	All	Boys	Girls	All	Boys	Girls	All	Boys	Girls	All
Anaemia												
Urban	44·6	33·2	39·0	1·3	2·0	1·6	4·9	1·0	3·2	3·6	1·4	2·6
Rural	40·6	45·6	43·0	1·0	2·3	1·6	11·3***	0·3	5·1	8·4**	0·7	4·2
All	43·2	37·4	40·3	1·2	2·1	1·6	6·5**	0·7	3·8	4·7*	1·2	3·0
Fe deficiency												
Urban				0·4*	4·2	2·3	4·0	3·7	3·9	2·7	3·9	3·3
Rural				0	3·1	1·4	0	3·9	2·3	0	3·7	2·1
All				0·3**	4·0	2·1	3·0	3·7	3·4	2·1	3·8	2·9
Vitamin A deficiency												
Urban				0	3·6	1·8	0·9††	2·7	1·7†	0·6††	3·1	1·8††
Rural				1·7*	11·0	6·0	10·0	5·2	7·2	7·6	6·3	6·9
All				0·3***	4·9	2·6	3·2	3·6	3·4	2·2	4·1	3·1
Vitamin D insufficiency												
Urban				7·3	15·7	11·4	22·8**	47·7	34·0	17·1**	34·8	25·3
Rural				0	7·8	3·5	14·9	43·0	31·3	10·4*	36·0	24·6
All				5·8	14·4	9·9	20·8***	46·0	33·2	15·6***	35·2	25·1

Percentage values were significantly different from girls of each age group based on complex sampling Pearson’s Chi-square: *p < 0.05, **p < 0.01 and ***p < 0.001.

Percentage values were significantly different from rural children based on complex sampling Pearson’s Chi-square: †*p* < 0.05, ††*p* < 0.01 and †††*p* < 0.001.

Prevalence of anaemia, Hb level: < 110 g/L (children < 5 years), <115 g/L (5–11 years) and < 120 g/L (children aged 12–14 years).

Fe deficiency, adjusted ferritin level: <12 µg/L (children< 5 years) and < 15 µg/L (children ≥ 5 years).

Vitamin A deficiency: mild (0·35 - 0·7 μmol/L) and severe (<0·35 μmol/L).

Vitamin D insufficiency: 25-hydroxyvitamin D < 50 nmol/L.

Mean macronutrient and micronutrient intakes by age groups are reported in Supplementary Tables 5 and 6, respectively. The percentage of children not achieving Malaysian RNI and the EAR of nutrients for all age groups are reported in Table 5 and Table 6, respectively. About half of the children (52·9 %) did not achieve energy recommendations. Majority of children did not achieve the RNI and EAR of Ca (RNI: 79·4 %, EAR: 70·4 %) and vitamin D (RNI: 94·8 %, EAR: 83·8 %). The highest proportions of children not achieving their Ca (RNI: 93·8 %, EAR: 87·7 %) and vitamin D (RNI: 98·5 %, EAR: 91·5 %) intakes were in the 7·0 to 12·9 years age group. Generally, there were significantly more girls than boys who did not achieve both the RNI and EAR for Fe, thiamine and vitamin A. When comparing children living in different areas, a higher percentage of rural children did not achieve the RNI of Fe, thiamine, riboflavin, vitamin C and vitamin A than urban children.

**Table 5.** Percentage of children not meeting the Malaysian recommended nutrient intake recommendations of nutrients by age groups and area of residence

	0·5–0·9 years			1·0–3·9 years			4·0–6·9 years			7·0–12·9 years			0·5–12·9 years		
	Boys	Girls	All	Boys	Girls	All	Boys	Girls	All	Boys	Girls	All	Boys	Girls	All
All															
Energy	42·3	27·5	36·4	26·1	22·1	24·2	42·1	46·1	44·0	73·1	69·3	71·3	53·8	51·9	52·9
Protein	13·2	8·1	11·2	0·2	0	0·1	0·2*	1·9	1·0	4·2*	7·9	6·0	2·8*	4·7	3·7
Ca	28·7#	13·6#	22·7#	50·0	53·8	51·8	82·4	87·6	84·9	92·5	95·2	93·8	77·6	81·4	79·4
Fe$	45·4	49·5	47·0	4·3	4·5	4·4	2·1	2·2	2·1	23·0***	35·3	29·0	14·9**	21·2	17·9
Thiamine	28·8	31·7	29·9	13·5	15·1	14·3	18·2	22·2	20·1	45·7*	54·3	49·8	31·2**	37·3	34·1
Riboflavin	27·8	23·2	26·0	3·0	4·8	3·9	6·6	8·8	7·6	33·9	36·0	34·9	20·3	22·3	21·2
Cobalamin#	68·0	57·8	63·9	28·6	30·1	29·3	24·9	28·7	26·7	58·6	64·0	61·3	44·3	48·0	46·1
Vitamin C	0	5·6	2·2	14·9	14·5	14·7	29·5	30·2	29·8	49·6	47·2	48·4	34·8	34·5	34·7
Vitamin A	10·0	14·1	11·6	17·8	20·9	19·2	27·9	28·1	28·0	37·0*	44·7	40·8	29·3*	34·5	31·8
Vitamin D	81·0#	77·0#	79·4#	88·9*	94·7	91·6	90·9	94·2	92·4	98·1	98·9	98·5	93·5*	96·1	94·8
Urban															
Energy	33·1	17·6	27·7	23·5	22·5	23·0	43·0	43·1	43·0	72·9	68·2	70·7	53·8	50·1	52·1
Protein	11·5	0	7·5	0·3	0	0·1	0	2·0	0·9	4·6	7·9	6·1	2·9	4·4	3·6
Ca	24·1#	0#	15·8#	48·7	52·7	50·7	80·4	86·6	83·3†	92·1	94·0	93·0	77·4	80·1	78·7
Fe$	44·1	33·0†	40·3	5·7	3·2	4·5	1·0†	2·9	1·9	22·0*	30·8††	26·0	14·6	17·6†††	16·0††
Thiamine	21·2	22·3	21·6	10·6	12·3	11·5†	15·4†	22·2	18·6	43·9*	52·0	47·5†	29·2*†	34·7††	31·7†††
Riboflavin	19·9	17·3	19·0	3·4	4·0	3·7	4·3*††	9·2	6·6	32·2	33·4	32·7†	19·1	20·2†	19·6††
Cobalamin#	70·4	56·7	65·6	28·5	29·1	28·8	24·8	28·3	26·4	57·9	63·3†	60·3	44·5	46·6	45·4
Vitamin C	0	0	0	13·9	11·5	12·7	28·2	28·0	28·1	48·3	43·5	46·1†	34·3	31·1††	32·8††
Vitamin A	7·0	5·0	6·3	14·3	18·4	16·3†	26·9	27·7	27·3	35·1*	42·7	38·5†	27·6	32·4†	29·8††
Vitamin D	80·5#	69·3#	76·6#	89·9	95·2	92·5	89·2†	94·7	91·8	98·4	98·7	98·5	93·6	96·1	94·8
Rural															
Energy	66·6	41·3	54·0	31·4	21·0	27·0	39·1*	55·9	47·1	74·1	71·7	72·8	53·8	56·5	55·1
Protein	17·8	19·4	18·6	0	0	0	0·9	1·7	1·3	2·9*	7·8	5·7	2·4*	5·6	4·0
Ca	40·6#	32·8#	36·7#	52·7	56·7	54·4	88·9	91·1	90·0	93·8	97·8	96·0	78·1	84·7	81·4
Fe$	48·7	72·8	60·7	2·2	7·1	4·3	5·6	0	3·0	26·3**	45·3	36·9	15·9***	30·0	23·0
Thiamine	48·7	44·9	46·8	19·4	22·6	20·8	27·3	22·2	24·9	51·7	59·4	56·0	36·9	43·9	40·4
Riboflavin	48·7	31·5	40·1	2·2	7·1	4·3	14·2	7·4	11·0	39·6	41·8	40·9	23·7	27·5	25·6
Cobalamin#	61·7	59·3	60·5	28·7	32·7	30·4	25·1	29·9	27·4	61·3	65·6	63·7	44·0	51·6	47·9
Vitamin C	0	13·4	6·7	17·1	22·5	19·4	33·7	37·2	35·3	53·9	55·2	54·7	36·2	42·9	39·6
Vitamin A	17·8	26·8	22·3	24·9	27·5	26·0	31·5	29·1	30·4	43·6	49·3	46·8	34·3	39·8	37·1
Vitamin D	82·0#	87·9#	85·0#	87·0	93·3	89·7	96·2	92·3	94·4	97·1*	99·2	98·3	93·2	96·1	94·7

Percentage values were significantly different from girls of each age group based on complex sampling Pearson’s Chi-square: *p < 0.05, **p < 0.01, ***p < 0.001.

Percentage values were significantly different from rural children based on complex sampling Pearson’s Chi-square: †*p* < 0.05, ††*p* < 0.01, †††*p* < 0.001.

# Values were compared with adequate intake.

$ Values were compared with 15 % bioavailability.

**Table 6.** Percentage of children not meeting the estimated average requirement of nutrients by age groups and area of residence

	0·5–0·9 years			1·0–3·9 years			4·0–6·9 years			7·0–12·9 years			0·5–12·9 years		
	Boys	Girls	All	Boys	Girls	All	Boys	Girls	All	Boys	Girls	All	Boys	Girls	All
All															
Protein	10·6	5·6	8·6	0·2	0	0·1	0	1·3	0·6	1·8*	4·3	3·0	1·4	2·7	2·0
Ca	–	–	–	27·0	31·1	29·0	72·0	75·4	73·6	85·1**	90·5	87·7	68·1*	73·0	70·4
Fe$	36·4	40·4	38·0	2·9	1·1	2·0	0·4	1·0	0·7	13·3*	20·4	16·7	8·9*	12·3	10·5
Thiamine	23·0	10·3	18·0	8·6	8·7	8·7	10·5	15·5	12·9	32·2**	42·7	37·3	21·4**	27·7	24·4
Riboflavin	16·8	5·6	12·3	2·0	0·6	1·3	3·5	4·5	4·0	20·0	19·1	19·6	11·9	11·1	11·5
Vitamin C	0	0	0	9·1	10·5	9·8	25·6	24·7	25·2	42·2	39·4	40·8	28·9	28·2	28·6
Vitamin A	0	5·6	2·2	6·5	8·6	7·5	15·5	12·0	13·8	18·8**	26·2	22·4	14·4*	18·3	16·2
Vitamin D	–	–	–	68·6	71·1	69·8	79·0	82·7	80·7	90·6	92·5	91·5	82·5	85·2	83·8
Urban															
Protein	11·5	0	7·5	0·3	0	0·1	0	1·7	0·8	1·9	4·9	3·3	1·6	2·9	2·2
Ca	–	–	–	23·5	28·0	25·8†	69·0†	72·7	70·8††	83·6	88·4†	85·8††	67·1	70·1††	68·5††
Fe$	31·7	33·0	32·1	4·3*	0·5	2·4	0·3	1·3	0·8	12·7*	18·3	15·2	8·8	10·4††	9·5†
Thiamine	16·3	3·8	12·0	6·5	4·5††	5·5††	8·3**†	16·4	12·1	30·3**	40·7	35·1†	19·5*†	25·4†	22·2†††
Riboflavin	11·5	0	7·5	1·9	0·8	1·3	2·2†	4·4	3·2	18·8	18·5	18·7	11·1	10·4	10·8
Vitamin C	0	0	0	6·8	9·8	8·3	24·3	22·9	23·7	42·3	36·6	39·7	28·8	26·0†	27·5
Vitamin A	0	0	0	4·3	5·8†	5·1††	14·1	11·9	13·1	19·4	24·3	21·7	14·2	16·3†	15·2†
Vitamin D	–	–	–	66·4	71·4	68·8	78·1	81·7	79·8	91·2	91·8	91·4	82·6	84·4	83·4
Rural															
Protein	8·1	13·4	10·7	0	0	0	0	0	0	1·2	2·8	2·1	0·9	2·2	1·6
Ca	–	–	–	34·1	39·4	36·4	81·7	83·8	82·7	90·1	94·9	92·8	71·0*	80·3	75·7
Fe$	48·7	50·9	49·8	0	2·6	1·1	0·9	0	0·5	15·2	25·1	20·8	9·3*	16·8	13·1
Thiamine	40·6	19·4	30·0	13·0	19·8	15·9	17·8	12·6	15·4	38·7	47·1	43·3	26·8	33·5	30·2
Riboflavin	30·8	13·4	22·1	2·2	0	1·3	7·7	4·8	6·3	23·9	20·6	22·0	14·4	12·9	13·6
Vitamin C	0	0	0	13·8	12·2	13·2	29·8	30·5	30·1	42·1	45·5	44·0	29·2	33·4	31·3
Vitamin A	0	13·4	6·7	10·9	15·9	13·1	19·8	12·1	16·2	16·8*	30·3	24·4	14·9*	23·0	19·0
Vitamin D	–	–	–	73·0	70·3	71·9	81·8	86·2	83·9	88·8	94·1	91·7	82·4	87·2	84·8

Percentage values were significantly different from girls of each age group based on complex sampling Pearson’s Chi-square: *p < 0.05, **p < 0.01, ***p < 0.001.

Percentage values were significantly different from rural children based on complex sampling Pearson’s Chi-square: †*p* < 0.05, ††*p* < 0.01, †††*p* < 0.001.

$ Values were compared with 15 % bioavailability.

## Discussion

The findings of the current SEANUTS II Malaysia highlight the prevalence of a triple burden of malnutrition among children aged 6 months to 12 years in Peninsular Malaysia. This necessitates an expansion of the previously reported double burden of malnutrition among Malaysian children to encompass the current status. Stunting and wasting problems are higher among children below 5 years, while the prevalence of obesity is higher in primary school-aged children (7·0–12·9 years old). Dietary data suggest that micronutrient intakes among Malaysian children are at a suboptimal level and particularly low in Ca and vitamin D. In addition, it was revealed that about a quarter of all children sampled had vitamin D insufficiency, which was significantly higher among girls than boys but not significantly different between urban and rural children.

Overall, the prevalences of thinness, overweight and obesity among children aged 6 months to 12 years were 6·7 %, 9·2 % and 8·8 % respectively. The prevalence of thinness reported in SEANUTS II Malaysia was slightly higher, while the prevalence of childhood obesity was lower compared to that reported in SEANUTS I Malaysia (thinness: 5·4 %, overweight: 9·8 % and obesity: 11·8 %)^(7)^. The current SEANUTS II Malaysia findings also show that overnutrition is more prevalent in children aged 7 to 12 years (overweight: 15·0 % and obese: 14·7 %) than children under 7 years old (overweight or obese: < 7 %). The Malaysian NHMS 2019 reported similar findings, whereby children aged 5 to 17 years had higher prevalences of overweight (15·0 %) and obesity (14·8 %), compared to those below 5 years (5·6 %)^(10)^. Besides, a recent secondary analysis of the NHMS data from 2006 to 2015 highlighted that the relative increase in the prevalence of overweight/obesity per year among children and adolescents aged 7–17 years in rural areas was much higher than their urban counterparts^(45)^. Thus, these findings should alert policymakers that overnutrition has become a public health issue of national concern regardless of area of residence.

In our study, boys, especially those aged 7 to 12 years, have a higher prevalence of obesity than girls. This finding is similar to that reported by NHMS 2019, in which the prevalence of obesity was 17·5 % for boys and 12·0 % for girls, among children aged 5 to 17 years^(10)^. The previous SEANUTS I Malaysia report also found higher obesity prevalence in boys, particularly among those residing in urban areas^(7)^. This pattern could stem from gender-based differences in food preferences and eating behaviours, with boys generally exhibiting higher consumption of protein-rich and calorie-dense foods, in contrast to girls who tend to favour the consumption of fruits and vegetables, which are less energy-dense^(46)^. This was also observed in the present study, where boys reported higher mean EI, particularly through their carbohydrate and protein intakes. Another reason may be that girls, starting as early as primary school age, are generally more concerned about their body size and body image. Our study incorporates a questionnaire on body image, with preliminary results indicating that a majority of children were dissatisfied with their body size (data not shown). Another local study also reported that more than two-thirds of girls aged 11 to 12 years (66 %) and more than half of the boys (52 %) had body size dissatisfaction^(47)^. Sex-related differences in body size and body image may therefore influence children’s eating practices and weight-related behaviours.

The prevalence of stunting in the present study among children aged 6 months to 12 years was 8·9 %, slightly higher than the percentage of children with stunting (8·4 %) reported in SEANUTS I Malaysia^(7)^. The prevalence of stunting was higher among children aged below 1 year (16·0 %), 1 to 3 years (14·4 %) and 4 to 6 years (8·9 %) than among children aged 7 to 12 years (5·6 %). Similar results were previously reported in SEANUTS I Malaysia, where the prevalence of stunting was also higher among children in younger age groups^(7)^. Upon investigating the aspect of area of residence, a distinct disparity was observed in the prevalence of stunting among girls aged 7–12 years residing in rural areas (11·3 %), as compared to their male counterparts (2·9 %). This discrepancy could be attributed to the higher prevalence of overweight/obesity among boys, which might indirectly contribute to a reduced prevalence of stunting within this group. The plausible explanation behind this phenomenon could be linked to the lower purchasing power of households within disadvantaged environments, a consequence of both the rising inflation rate^(48)^ and the widening of income inequality between lower- and higher-income populations (Gini coefficient in 2014: 0·401 *v*. 2019: 0·407)^(13)^. Consequently, this culminates in inadequate energy and nutrient intakes among children in households with lower socio-economic status.

Among children below 5 years, the present study found that prevalence of stunting was 13·8 %. NHMS 2019 had also reported a higher prevalence of stunting among children below 5 years (21·8 %) and a lower prevalence among children and adolescents aged 5 to 17 years (12·7 %)^(10)^. The prevalences of wasting and underweight among children below 5 years in the present study were 6·2 % and 11·4 %, respectively. Our findings were lower than the prevalence reported in NHMS 2019. However, it is worth noting that these prevalences have both decreased and increased over the years. Based on NHMS data, the prevalence of wasting decreased from 12·4 % in 2011 to 8·1 % in 2015 and increased again to 9·7 % in 2019^(10–12)^. On the other hand, according to NHMS data, prevalence of underweight increased from 11·6 % in 2011 to 12·4 % in 2015 and further increased to 14·1 % in 2019^(10,11)^.

The differences in findings may be due to the sampling frame used as SEANUTS II involved only four main regions of Peninsular Malaysia, while the NHMS surveys recruited children from all states and federal territories in Malaysia. Additionally, the NHMS reported overall prevalences for children and adolescents up to 17 years of age, while SEANUTS II included children only up to 12 years of age. The overall representation of Malaysian children from this study was limited to only Peninsular Malaysia, as we were only able to conduct our study with Malaysian children from this part of the country. We had to terminate data collection in East Malaysia, which comprised the states of Sabah and Sarawak, because of the COVID-19 pandemic. However, in a study conducted in 2018 in Sabah, the prevalences of underweight, stunting and wasting among children below 5 years old were reported to be high at 34·7 %, 33·3 % and 10·0 %, respectively^(49)^. SEANUTS II’s inability to include children in Sabah and Sarawak has resulted in the exclusion of their undernutrition problems from being considered in the study.

When the current data on anaemia, Fe deficiency, vitamin A deficiency and vitamin D insufficiency are compared with that of SEANUTS I Malaysia, the current prevalences are observably lower than from a decade ago, at 3·0 % *v*. 6·6 %, 2·9 % *v*. 4·4 %, 3·1 % *v*. 4·4 % and 25·1 % *v*. 47·5 %, respectively^(7)^. However, SEANUTS II Malaysia found a high prevalence of anaemia (40·3 %) among children below 4 years old, which indicates that anaemia among young children in Peninsular Malaysia was a problem of severe public health significance. As anaemia may affect cognitive development, growth rate and immunity of children^(50)^, this is a matter that requires urgent attention and action. A local study conducted in Penang, Malaysia, reported that about 22·3 % of children aged 6 months to 15 years were anaemic^(50)^. In Malaysia, NHMS 2019 reported that approximately 21 % of the population aged 15 years and above is anaemic, with an even higher prevalence (30 %) among women of reproductive age^(10)^. Moreover, while the NHMS 2019 reported that about 20·5 % of Malaysian adolescents aged 15 to 19 years were anaemic^(10)^, it did not assess the prevalence of anaemia among children younger than 15 years old. Our study found that boys in the 7-to-12·9-year age group had a higher prevalence of anaemia compared to girls. The closest age group available for comparison is data from a local adolescent longitudinal study, which, in contrast to our study, reported significantly higher prevalence of anaemia among girls than boys at ages 13, 15 and 17 years^(51)^. This is understandable as most adolescent girls would have attained menarche or may even have regular menses by the age of 13 years. It is also worth noting that the prevalence of anaemia among indigenous *Bumiputra* children from Sabah and Sarawak and *Orang Asli* children in Peninsular Malaysia were particularly high, as recorded in the SEANUTS I Malaysia study^(52)^. The current lower prevalence of anaemia, compared to previous studies, could be due to the lack of indigenous children’s samples, especially from Sabah and Sarawak.

SEANUTS II Malaysia determined that the present prevalence of children with vitamin D insufficiency (25·1 %) was only about half the proportion of children with vitamin D insufficiency (47·5 %) 10 years ago, as determined by SEANUTS I Malaysia^(7)^. Our current finding is also lower than the findings reported for children in SEANUTS I counterpart countries, Indonesia, Thailand and Vietnam (33·7 % – 48·2 %)^(53)^. This finding is encouraging as it implies significant improvement in the vitamin D status of Malaysian children, particularly among boys. The lower prevalence of vitamin D insufficiency among preschoolers may be due to higher consumption of vitamin D-fortified foods, including fortified milk and dairy products, which are more commonly consumed by those below 7 years old^(53)^. Similar to SEANUTS I Malaysia, the present study shows a higher percentage of girls with vitamin D insufficiency^(7)^. This finding is consistent with other local studies conducted among primary schoolchildren, adolescents and adults^(54)^. This may be due to girls wearing skin-covering clothes, which exposes less body surface area to the sun compared with boys^(55)^. Muslim girls are likely to have a greater sections of their bodies covered with clothes, for example, wearing hijabs to cover their heads and necks^(52)^. Girls are also more likely to spend less time in outdoor play as well as using sunscreen when outdoors^(55)^. Other factors that may be related to vitamin D insufficiency are skin colour, dietary intake (including vitamin D supplementation), obesity and diseases related to fat malabsorption^(54)^. Further studies involving national data are needed to determine the predictive factors of vitamin D insufficiency.

The present study also notes a major concern in the low dietary intake of Ca and vitamin D, as a majority of children did not meet nutrient intake guidelines (Ca RNI: 79·4 %, EAR: 70·4 %; vitamin D RNI: 94·8 %, EAR: 83·8 %). These percentages are much higher, compared to the percentages of not achieving RNI of Ca (urban: 49·9 %; rural: 49·1 %) and vitamin D (urban: 48·3 %; rural: 50·1 %) reported in SEANUTS I Malaysia^(7)^. However, it is worth noting that different versions of RNI were used in SEANUTS I Malaysia and SEANUTS II Malaysia, following the revision of the RNI in 2017, whereby the recommended intake for Ca and vitamin D increased by an average of 200–300 mg and 10 µg, respectively^(56)^. A similar finding was reported by a national study conducted among Malaysian adolescents aged 13 to 17 years^(57)^ where high proportions of adolescents (98·8 % and 89·4 %, respectively) consumed less than 75 % of the recommended levels of vitamin D and Ca based on RNI 2017. On the contrary, data of vitamin D showed that only a quarter of children had vitamin D insufficiency, whereas more than 90 % of children did not meet the RNI for vitamin D intake. A possible reason for the discrepancy between blood and dietary intake data may be due to limitations related to the food composition database. Using the vitamin D food databases from other countries (UK, USDA) may not provide accurate estimates of the vitamin D content of local foods. Another reason could be due to bias during dietary recall. Moreover, serum vitamin D status represents total exposure including those from intake as well as from de novo synthesis of vitamin D in the skin. Thus, it seems possible that some children may have sufficient vitamin D production, despite a low vitamin D intake. Notwithstanding these limitations, the high percentages of children not meeting the Ca and vitamin D dietary intake are still alarming. Thus, all relevant parties (i.e. government agencies, private sectors, food industries and other family-focused agencies) should strengthen strategies and programmes that promote Ca and vitamin D intake, while encouraging outdoor play.

The main limitation of this study was the sampling, as the population of children we studied lacked representativeness for the whole Malaysia, due to the termination of data collection in Sabah and Sarawak, with the advent of COVID-19 and the implementation of the subsequent movement control orders. However, the sample used in our study is representative of children aged 6 months to 12 years in Peninsular Malaysia. Moreover, the data were weighted and analysed with Complex Sampling Analyses to better represent Malaysian children pertaining to their current nutritional status, nutritional biomarkers and dietary intake.

Another study limitation is related to the reliance on a single administration of 24-h recall for dietary assessment. While a single 24-h diet recall data provide mean nutrient intake, it does not capture day-to-day variability in the diet, and thus, precludes estimation of usual dietary distribution. In addition, under-reporting is inherent to the 24-h diet recall method, and this is made even more likely when dietary intake data were proxy-reported by parents or caregivers of children aged 6 months to 9 years as eating occasions may occur in the absence of parents (e.g. in childcare centres or schools). Moreover, it is possible that omitting the data on dietary supplements from the analysis could potentially have an influence on the overall nutrient intakes among children.

SEANUTS II Malaysia presents the latest comprehensive national nutrition data of Malaysian children aged 6 months to 12 years as a follow-up to SEANUTS I Malaysia, which was conducted a decade earlier. Taking into consideration the sample size and regions covered, we consider the data collected from the children to be representative of the current nutritional status of Malaysian children, particularly those residing in Peninsular Malaysia. One of the main strengths of SEANUTS II is the standardised protocols employed in this multi-centre survey, which render the findings from all four countries involved, namely Malaysia, Indonesia, Thailand and Vietnam, comparable, thus, providing a comprehensive picture of the nutritional status and dietary intakes of Southeast Asian children. The survey was also conducted in a manner that encouraged harmonising of methods and sharing of information; hence, we anticipate that all the counterpart countries will be able to act accordingly based on the current SEANUTS II findings.

### Conclusion

The findings from SEANUTS II Malaysia confirm that the triple burden of malnutrition exists in Malaysia, with the prevalence of overnutrition being higher than undernutrition. Notably, there were also suboptimal levels of micronutrients. The prevalence of anaemia was of severe public health significance among children below 4 years and vitamin D insufficiency was high, especially among older girls. Dietary intake data revealed such major concerns as the low Ca and vitamin D intakes among the children. SEANUTS II’s comprehensive and updated data are anticipated to offer a valuable point of reference for the NPANM III review, providing contemporary insights for policy-making, which includes strategic planning, the setting of targets for action plans, and implementing programmes aimed at improving nutritional status and dietary intake, as well as addressing the triple burden of malnutrition among Malaysian children.

## Supporting information

Poh et al. supplementary material Poh et al. supplementary material

## Supplementary material

To view supplementary material for this article, please visit https://doi.org/10.1017/S1368980023002239
