# Supplementary material for: Triple burden of malnutrition among Malaysian children aged 6 months to 12 years: current findings from SEANUTS II Malaysia
Source: Public Health Nutr. 2023 Nov 7;27(1):e151. doi: 10.1017/S1368980023002239 (PMC11617412; doi:10.1017/S1368980023002239)
Supplement: Poh et al. supplementary material [file S1368980023002239sup001.docx]

SUPPLEMENTARY TABLE 1 Sociodemographic characteristics of children by home-based and school-based approaches

|  | **Home-based approach** | | | **School-based approach** | | | **All** | | |
| --- | --- | --- | --- | --- | --- | --- | --- | --- | --- |
|  | **n** | **%** | **95% CI** | **n** | **%** | **95% CI** | **n** | **%** | **95% CI** |
| **Sex** |  |  |  |  |  |  |  |  |  |
| Boys | 731 | 51.7 | 48.5-54.8 | 701 | 51.7 | 48.6-54.8 | 1432 | 51.7 | 49.5-53.9 |
| Girls | 727 | 48.3 | 45.2-51.5 | 830 | 48.3 | 45.2-51.4 | 1557 | 48.3 | 46.1-50.5 |
| **Area of residence** |  |  |  |  |  |  |  |  |  |
| Urban | 1040 | 72.9 | 70.1-75.5 | 1063 | 73.8 | 71.1-76.4 | 2103 | 73.3 | 71.4-75.2 |
| Rural | 418 | 27.1 | 24.5-29.9 | 468 | 26.2 | 23.6-28.9 | 886 | 26.7 | 24.8-28.6 |
| **Ethnicities** |  |  |  |  |  |  |  |  |  |
| Malay | 903 | 77.1 | 74.6-79.4 | 889 | 71.7 | 69.0-74.3 | 1792 | 74.4 | 72.5-76.2 |
| Chinese | 410 | 14.0 | 12.6-15.6 | 461 | 17.5 | 15.7-19.4 | 871 | 15.8 | 14.6-17.0 |
| Indian | 118 | 5.4 | 4.4-6.7 | 162 | 7.0 | 5.8-8.4 | 280 | 6.2 | 5.4-7.1 |
| Other races | 27 | 3.5 | 2.0-5.9 | 19 | 3.8 | 2.2-6.4 | 46 | 3.6 | 2.5-5.3 |
| **Paternal education** |  |  |  |  |  |  |  |  |  |
| Non-schooling/Primary | 66 | 3.8 | 2.8-5.1 | 74 | 4.7 | 3.3-6.5 | 140 | 4.2 | 3.4-5.3 |
| Secondary | 747 | 54.0 | 50.9-57.1 | 751 | 51.1 | 47.8-54.3 | 1498 | 52.5 | 50.3-54.8 |
| Tertiary | 582 | 42.2 | 39.1-45.3 | 613 | 44.3 | 41.1-47.5 | 1195 | 43.2 | 41.0-45.5 |
| **Maternal education** |  |  |  |  |  |  |  |  |  |
| Non-schooling/Primary | 63 | 3.8 | 2.8-5.1 | 53 | 3.3 | 2.2-4.7 | 116 | 3.5 | 2.8-4.4 |
| Secondary | 661 | 45.9 | 42.8-49.1 | 738 | 48.3 | 45.2-51.5 | 1399 | 47.1 | 44.9-49.4 |
| Tertiary | 724 | 50.3 | 47.2-53.4 | 720 | 48.4 | 45.3-51.5 | 1444 | 49.4 | 47.1-51.6 |
| **Household income** |  |  |  |  |  |  |  |  |  |
| Low (< RM 4850) | 860 | 63.1 | 60.1-66.0 | 831 | 56.2 | 53.1-59.4 | 1691 | 59.7 | 57.5-61.8 |
| Medium (RM 4850-RM 10959) | 482 | 31.1 | 28.3-34.0 | 519 | 32.8 | 30.0-35.8 | 1001 | 32.0 | 30.0-34.0 |
| High (≥RM 10960) | 96 | 5.8 | 4.6-7.2 | 144 | 10.9 | 8.9-13.4 | 240 | 8.4 | 7.1-9.8 |

n=unweighted count, statistical analysis was performed using complex sampling Pearson Chi-square and no significant difference was found in the sociodemographic characteristics between

home-based and school-based recruitment approaches

GBP 1 = MYR 5.17 (as of 3 October 2022)

Supplementary TABLE 2 Percentage (%) of stunted, wasted, underweight, thin, normal weight, overweight, and obese children aged 0.5 to 12.9 years

|  | 0.5-4.9 years | | | 5.0-12.9 years | | |
| --- | --- | --- | --- | --- | --- | --- |
|  | **Boys** | **Girls** | **All** | **Boys** | **Girls** | **All** |
| All |  |  |  |  |  |  |
| Stunted | 14.3 | 13.2 | 13.8 | 5.2 | 7.2 | 6.2 |
| Underweight^α^ | 11.8 | 10.9 | 11.4 | - | - | - |
| Wasted ^α^ | 5.9 | 6.5 | 6.2 | - | - | - |
| Wasting/thinness | 6.0 | 5.5 | 5.8 | 6.9 | 7.7 | 7.3 |
| Overweight | 2.4 | 1.9 | 2.2 | 13.1 | 13.1 | 13.1 |
| Obese | 0.9 | 0.6 | 0.8 | 16.1** | 10.3 | 13.3 |
| Urban |  |  |  |  |  |  |
| Stunted | 14.2 | 13.5 | 13.9 | 5.3 | 5.1†† | 5.2†† |
| Underweight^α^ | 11.1 | 10.7 | 10.9 | - | - | - |
| Wasted^α^ | 5.1 | 6.1 | 5.6 | - | - | - |
| Wasting/thinness | 5.0 | 5.3 | 5.2 | 6.8 | 7.5 | 7.1 |
| Overweight | 3.1 | 1.8 | 2.4 | 13.7 | 12.9 | 13.3 |
| Obese | 0.6 | 0.4 | 0.5 | 14.9** | 9.5 | 12.4 |
| Rural |  |  |  |  |  |  |
| Stunted | 14.6 | 12.3 | 13.6 | 5.2* | 12.2 | 9.1 |
| Underweight^α^ | 13.4 | 11.3 | 12.4 | - | - | - |
| Wasted^α^ | 7.7 | 7.7 | 7.7 | - | - | - |
| Wasting/thinness | 8.2 | 6.0 | 7.2 | 7.3 | 8.1 | 7.7 |
| Overweight | 1.0 | 2.3 | 1.6 | 11.3 | 13.7 | 12.7 |
| Obese | 1.6 | 1.1 | 1.4 | 20.2* | 12.4 | 15.9 |

^α^The data analyses involved children below 5 years old only.

Percentage values were significantly different from girls of each age group based on complex sampling Pearson Chi-square: **p*<0.05, ***p*<0.01, ****p*<0.001.

Percentage values were significantly different from rural children based on complex sampling Pearson Chi-square: †*p*<0.05, ††*p*<0.01, †††*p*<0.001.

Definition of nutritional status: stunted: height-for-age (HAZ) <-2 SD from the median; underweight (under 5 years only): weight-for-age (WAZ) <-2 SD from the median;

wasted (under 5 years only): weight-for-height (WHZ) <-2 SD from the median; wasting (under 5 years): BMI-for-age (BAZ) <-2 SD from the median;

thinness (5-12 years only): BMI-for-age (BAZ) <-2 SD from the median; overweight: BMI-for-age (BAZ) >2 SD (< 5 years) and >1 SD (5-12 years) from the median;

obese: BMI-for-age (BAZ) >3 SD (< 5 years) and >2 SD (5-12 years) from the median

Supplementary TABLE 3 Anthropometric characteristics of boys and girls by age group

|  | 0.5-0.9 years | | | | | | 1.0-3.9 years | | | | | | 4.0-6.9 years | | | | | | 7.0-12.9 years | | | | | |
| --- | --- | --- | --- | --- | --- | --- | --- | --- | --- | --- | --- | --- | --- | --- | --- | --- | --- | --- | --- | --- | --- | --- | --- | --- |
|  | **Boys** | | **Girls** | | **All** | | **Boys** | | **Girls** | | **All** | | **Boys** | | **Girls** | | **All** | | **Boys** | | **Girls** | | **All** | |
|  | **Mean** | **SE** | **Mean** | **SE** | **Mean** | **SE** | **Mean** | **SE** | **Mean** | **SE** | **Mean** | **SE** | **Mean** | **SE** | **Mean** | **SE** | **Mean** | **SE** | **Mean** | **SE** | **Mean** | **SE** | **Mean** | **SE** |
| All |  |  |  |  |  |  |  |  |  |  |  |  |  |  |  |  |  |  |  |  |  |  |  |  |
| Age (years) | 0.79 | 0.03 | 0.80 | 0.02 | 0.80 | 0.02 | 2.57 | 0.06 | 2.53 | 0.06 | 2.55 | 0.04 | 5.49 | 0.05 | 5.49 | 0.05 | 5.49 | 0.04 | 9.91 | 0.09 | 9.92 | 0.08 | 9.91 | 0.06 |
| Weight (kg) | 8.3* | 0.2 | 7.7 | 0.2 | 8.0 | 0.1 | 12.3** | 0.1 | 11.8 | 0.1 | 12.1 | 0.1 | 18.7* | 0.2 | 18.0 | 0.2 | 18.4 | 0.1 | 33.7 | 0.5 | 33.2 | 0.5 | 33.5 | 0.3 |
| Height (cm) | 71.2* | 0.5 | 69.4 | 0.6 | 70.3 | 0.4 | 88.6* | 0.2 | 87.6 | 0.3 | 88.1 | 0.2 | 109.8** | 0.3 | 108.7 | 0.3 | 109.2 | 0.2 | 135.2 | 0.3 | 135.7 | 0.3 | 135.4 | 0.2 |
| BMI (kg/m^2^) | 16.2 | 0.3 | 15.9 | 0.2 | 16.0 | 0.2 | 15.6* | 0.1 | 15.3 | 0.1 | 15.4 | 0.1 | 15.3 | 0.1 | 15.1 | 0.1 | 15.2 | 0.1 | 18.0 | 0.2 | 17.5 | 0.2 | 17.7 | 0.1 |
| HAZ | -0.59 | 0.25 | -0.56 | 0.25 | -0.57 | 0.17 | -0.93 | 0.07 | -0.82 | 0.09 | -0.87 | 0.05 | -0.63 | 0.06 | -0.70 | 0.05 | -0.66 | 0.04 | -0.39 | 0.05 | -0.41 | 0.05 | -0.4 | 0.03 |
| WAZ | -0.87 | 0.24 | -0.76 | 0.21 | -0.81 | 0.16 | -0.73 | 0.07 | -0.68 | 0.08 | -0.70 | 0.06 | -0.48 | 0.07 | -0.63 | 0.07 | -0.55 | 0.05 | -0.18 | 0.09 | -0.29 | 0.08 | -0.24 | 0.06 |
| WHZ | -0.70 | 0.22 | -0.57 | 0.16 | -0.63 | 0.14 | -0.34 | 0.08 | -0.34 | 0.08 | -0.34 | 0.06 | -0.21 | 0.12 | -0.49 | 0.13 | -0.35 | 0.09 | - | - | - | - | - | - |
| BAZ | -0.71 | 0.22 | -0.60 | 0.16 | -0.65 | 0.14 | -0.23 | 0.08 | -0.24 | 0.08 | -0.23 | 0.05 | -0.15 | 0.07 | -0.31 | 0.07 | -0.23 | 0.05 | 0.22** | 0.08 | -0.04 | 0.06 | 0.09 | 0.05 |
| Urban |  |  |  |  |  |  |  |  |  |  |  |  |  |  |  |  |  |  |  |  |  |  |  |  |
| Age (years) | 0.79 | 0.04 | 0.81 | 0.02 | 0.80 | 0.02 | 2.62 | 0.07 | 2.55 | 0.07 | 2.58 | 0.05 | 5.52 | 0.06 | 5.46 | 0.06 | 5.49 | 0.04 | 9.83 | 0.10 | 9.84 | 0.09 | 9.84† | 0.07 |
| Weight (kg) | 8.0 | 0.2 | 7.6 | 0.2 | 7.8 | 0.2 | 12.4* | 0.1 | 11.9 | 0.2 | 12.1 | 0.1 | 18.7 | 0.2 | 18.2 | 0.2 | 18.5 | 0.2 | 33.5 | 0.5 | 33.0 | 0.5 | 33.3 | 0.4 |
| Height (cm) | 71.3 | 0.7 | 69.5 | 0.8 | 70.5 | 0.6 | 88.6 | 0.3 | 87.8 | 0.4 | 88.2 | 0.2 | 109.9* | 0.3 | 109.0† | 0.3 | 109.5† | 0.2 | 135.3 | 0.4 | 136.1 | 0.3 | 135.7 | 0.3 |
| BMI (kg/m^2^) | 15.8†† | 0.3 | 15.6 | 0.2 | 15.7†† | 0.2 | 15.6* | 0.1 | 15.3 | 0.1 | 15.4 | 0.1 | 15.3 | 0.1 | 15.1 | 0.1 | 15.2 | 0.1 | 17.9* | 0.2 | 17.3 | 0.2 | 17.6 | 0.1 |
| HAZ | -0.56 | 0.32 | -0.51 | 0.34 | -0.54 | 0.23 | -0.92 | 0.08 | -0.76 | 0.11 | -0.84 | 0.07 | -0.59 | 0.07 | -0.64† | 0.06 | -0.62† | 0.05 | -0.36 | 0.06 | -0.34† | 0.05 | -0.35† | 0.04 |
| WAZ | -1.09 | 0.28 | -0.86 | 0.25 | -0.98 | 0.19 | -0.71 | 0.09 | -0.64 | 0.10 | -0.68 | 0.07 | -0.47 | 0.09 | -0.57 | 0.08 | -0.52 | 0.06 | -0.17 | 0.11 | -0.30 | 0.10 | -0.23 | 0.07 |
| WHZ | -1.02† | 0.22 | -0.71 | 0.14 | -0.88†† | 0.14 | -0.31 | 0.09 | -0.34 | 0.09 | -0.33 | 0.06 | -0.24 | 0.14 | -0.51 | 0.15 | -0.37 | 0.10 | - | - | - | - | - | - |
| BAZ | -1.03†† | 0.22 | -0.76 | 0.14 | -0.91†† | 0.14 | -0.20 | 0.09 | -0.24 | 0.09 | -0.22 | 0.06 | -0.17 | 0.09 | -0.29 | 0.08 | -0.23 | 0.06 | 0.20* | 0.09 | -0.09 | 0.07 | 0.07 | 0.06 |
| Rural |  |  |  |  |  |  |  |  |  |  |  |  |  |  |  |  |  |  |  |  |  |  |  |  |
| Age (years) | 0.79 | 0.03 | 0.8 | 0.05 | 0.79 | 0.03 | 2.47 | 0.1 | 2.49 | 0.09 | 2.48 | 0.07 | 5.40 | 0.08 | 5.58 | 0.1 | 5.49 | 0.06 | 10.15 | 0.19 | 10.10 | 0.17 | 10.12 | 0.12 |
| Weight (kg) | 8.8* | 0.3 | 7.8 | 0.3 | 8.3 | 0.3 | 12.1 | 0.2 | 11.8 | 0.2 | 12.0 | 0.1 | 18.6* | 0.3 | 17.5 | 0.4 | 18.0 | 0.3 | 34.2 | 1.0 | 33.9 | 1.0 | 34.0 | 0.7 |
| Height (cm) | 71.2* | 0.7 | 69.2 | 0.7 | 70.2 | 0.5 | 88.5* | 0.4 | 87.1 | 0.4 | 87.9 | 0.3 | 109.2* | 0.4 | 107.7 | 0.4 | 108.5 | 0.3 | 134.7 | 0.5 | 134.8 | 0.6 | 134.8 | 0.4 |
| BMI (kg/m^2^) | 17.2 | 0.4 | 16.4 | 0.5 | 16.8 | 0.4 | 15.5 | 0.2 | 15.3 | 0.2 | 15.5 | 0.1 | 15.4 | 0.2 | 14.9 | 0.2 | 15.2 | 0.1 | 18.3 | 0.4 | 18.0 | 0.4 | 18.1 | 0.3 |
| HAZ | -0.66 | 0.32 | -0.65 | 0.27 | -0.66 | 0.21 | -0.94 | 0.12 | -0.98 | 0.12 | -0.96 | 0.09 | -0.75 | 0.09 | -0.88 | 0.09 | -0.82 | 0.06 | -0.47 | 0.08 | -0.57 | 0.10 | -0.52 | 0.06 |
| WAZ | -0.35 | 0.37 | -0.57 | 0.34 | -0.46 | 0.26 | -0.79 | 0.14 | -0.76 | 0.14 | -0.78 | 0.10 | -0.52 | 0.13 | -0.80 | 0.13 | -0.66 | 0.09 | -0.26 | 0.19 | -0.25 | 0.16 | -0.26 | 0.12 |
| WHZ | 0.04 | 0.35 | -0.27 | 0.33 | -0.11 | 0.25 | -0.41 | 0.15 | -0.34 | 0.16 | -0.38 | 0.11 | -0.09 | 0.23 | -0.42 | 0.23 | -0.25 | 0.17 | - | - | - | - | - | - |
| BAZ | 0.05 | 0.34 | -0.27 | 0.33 | -0.11 | 0.25 | -0.29 | 0.15 | -0.24 | 0.16 | -0.27 | 0.11 | -0.08 | 0.14 | -0.37 | 0.12 | -0.22 | 0.09 | 0.29 | 0.16 | 0.07 | 0.13 | 0.16 | 0.10 |

Abbreviations: SE, standard error; BMI, body mass index; HAZ, height-for-age z score; WAZ, weight-for-age z score; WHZ, weight-for-height z score; BAZ, BMI-for-age z score.

Mean values were significantly different from girls of each age group based on complex sampling ANCOVA after adjusted for age: **p*<0.05, ***p*<0.01, ****p*<0.001.

Mean values were significantly different from rural children based on complex sampling ANCOVA after adjusted for age: †*p*<0.05, ††*p*<0.01, †††*p*<0.001.

Supplementary TABLE 4 Nutritional biomarker of boys and girls aged 0.5-3.9 years

| Haemoglobin (g/L) | Boys | | Girls | | All | |
| --- | --- | --- | --- | --- | --- | --- |
|  | **Mean** | **SE** | **Mean** | **SE** | **Mean** | **SE** |
| All | 111.0 | 1.4 | 110.5 | 1.2 | 110.7 | 0.9 |
| Urban | 110.4 | 1.5 | 112.9 | 1.5 | 111.6 | 1.1 |
| Rural | 110.6 | 2.0 | 107.2 | 2.6 | 109.0 | 1.6 |

Abbreviations: SE, standard error.

Supplementary TABLE 5 Macronutrient intake by age group in boys and girls

|  | 0.5-0.9 years | | | | | | 1.0-3.9 years | | | | | | 4.0-6.9 years | | | | | | 7.0-12.9 years | | | | | |
| --- | --- | --- | --- | --- | --- | --- | --- | --- | --- | --- | --- | --- | --- | --- | --- | --- | --- | --- | --- | --- | --- | --- | --- | --- |
|  | **Boys** | | **Girls** | | **All** | | **Boys** | | **Girls** | | **All** | | **Boys** | | **Girls** | | **All** | | **Boys** | | **Girls** | | **All** | |
|  | **Mean** | **SE** | **Mean** | **SE** | **Mean** | **SE** | **Mean** | **SE** | **Mean** | **SE** | **Mean** | **SE** | **Mean** | **SE** | **Mean** | **SE** | **Mean** | **SE** | **Mean** | **SE** | **Mean** | **SE** | **Mean** | **SE** |
| All |  |  |  |  |  |  |  |  |  |  |  |  |  |  |  |  |  |  |  |  |  |  |  |  |
| Energy (kcal) | 721 | 28 | 737 | 31 | 729 | 21 | 1219* | 28 | 1142 | 21 | 1181 | 17 | 1434*** | 26 | 1290 | 25 | 1362 | 18 | 1616*** | 25 | 1465 | 23 | 1540 | 17 |
| Carbohydrate (g) | 85.3 | 4.1 | 93.0 | 5.5 | 89.2 | 3.5 | 167.1* | 4.9 | 153.4 | 3.3 | 160.2 | 3.0 | 193.0** | 4.0 | 178.0 | 3.7 | 185.5 | 2.7 | 218.0*** | 3.4 | 200.1 | 3.1 | 209.1 | 2.3 |
| CHO (%TEI) | 46.8 | 0.9 | 49.6 | 1.4 | 48.2 | 0.8 | 54.4 | 0.6 | 53.7 | 0.5 | 54.1 | 0.4 | 53.9* | 0.4 | 55.4 | 0.5 | 54.7 | 0.3 | 54.3 | 0.4 | 54.9 | 0.3 | 54.6 | 0.2 |
| Protein (g) | 22.1 | 1.8 | 20.0 | 1.1 | 21.0 | 1.1 | 43.1 | 1.2 | 42.3 | 0.9 | 42.7 | 0.7 | 52.3*** | 1.0 | 46.5 | 1.1 | 49.4 | 0.7 | 61.3*** | 1.2 | 54.0 | 1.1 | 57.6 | 0.8 |
| PRO (%TEI) | 11.9 | 0.9 | 10.9 | 0.8 | 11.4 | 0.6 | 14.2 | 0.3 | 14.9 | 0.3 | 14.5 | 0.2 | 14.9 | 0.2 | 14.5 | 0.2 | 14.7 | 0.2 | 15.2 | 0.2 | 14.8 | 0.2 | 15.0 | 0.1 |
| Fat (g) | 32.7 | 1.0 | 32.0 | 1.3 | 32.3 | 0.8 | 42.0 | 1.0 | 39.9 | 1.0 | 41.0 | 0.7 | 50.1*** | 1.1 | 43.4 | 1.0 | 46.8 | 0.8 | 55.3*** | 1.1 | 49.9 | 1.0 | 52.6 | 0.8 |
| FAT (%TEI) | 41.7 | 1.1 | 39.9 | 1.3 | 40.8 | 0.8 | 31.3 | 0.5 | 31.5 | 0.4 | 31.4 | 0.3 | 31.2* | 0.4 | 30.0 | 0.4 | 30.6 | 0.3 | 30.4 | 0.3 | 30.2 | 0.3 | 30.3 | 0.2 |
| Urban |  |  |  |  |  |  |  |  |  |  |  |  |  |  |  |  |  |  |  |  |  |  |  |  |
| Energy (kcal) | 734 | 30 | 789† | 33 | 753 | 24 | 1218* | 33 | 1131 | 24 | 1175 | 21 | 1444** | 30 | 1302 | 28 | 1377 | 21 | 1622*** | 29 | 1471 | 28 | 1553 | 21 |
| Carbohydrate (g) | 86.0* | 4.6 | 104.3†† | 5.7 | 92.4 | 4.1 | 168.1* | 6.2 | 152.3 | 3.9 | 160.3 | 3.8 | 195.5** | 4.6 | 179.1 | 4.1 | 187.8 | 3.2 | 217.1* | 4.0 | 202.9 | 3.9 | 210.6 | 2.8 |
| CHO (%TEI) | 46.4** | 1.1 | 52.6†† | 1.4 | 48.5 | 1.1 | 54.9 | 0.7 | 53.7 | 0.6 | 54.4 | 0.5 | 54.2 | 0.5 | 55.3 | 0.5 | 54.7 | 0.4 | 53.8**†† | 0.4 | 55.4† | 0.4 | 54.5 | 0.3 |
| Protein (g) | 23.3 | 2.1 | 20.8 | 1.5 | 22.5 | 1.5 | 42.3 | 1.3 | 42.8 | 1.1 | 42.5 | 0.9 | 52.0** | 1.2 | 47.4 | 1.2 | 49.9 | 0.9 | 61.7*** | 1.4 | 52.4† | 1.2 | 57.4 | 1.0 |
| PRO (%TEI) | 12.6 | 1.1 | 10.5 | 0.6 | 11.9 | 0.8 | 14.0** | 0.3 | 15.2† | 0.3 | 14.6 | 0.2 | 14.6 | 0.3 | 14.6 | 0.3 | 14.6 | 0.2 | 15.3** | 0.2 | 14.4††† | 0.2 | 14.9† | 0.2 |
| Fat (g) | 33.1 | 1.1 | 32.1 | 1.7 | 32.8 | 1.0 | 41.7 | 1.2 | 39.0 | 1.0 | 40.4 | 0.8 | 50.4*** | 1.3 | 43.9 | 1.2 | 47.3 | 0.9 | 56.2** | 1.4 | 50.1 | 1.2 | 53.4 | 0.9 |
| FAT (%TEI) | 41.2* | 1.3 | 37.1†† | 1.6 | 39.8† | 1.1 | 31.1 | 0.6 | 31.1 | 0.5 | 31.1 | 0.4 | 31.1* | 0.4 | 29.9 | 0.4 | 30.6 | 0.3 | 30.9†† | 0.4 | 30.3 | 0.4 | 30.6† | 0.3 |
| Rural |  |  |  |  |  |  |  |  |  |  |  |  |  |  |  |  |  |  |  |  |  |  |  |  |
| Energy (kcal) | 687 | 61 | 665 | 44 | 676 | 38 | 1223 | 49 | 1172 | 44 | 1202 | 34 | 1401* | 49 | 1252 | 53 | 1331 | 36 | 1595* | 47 | 1453 | 41 | 1516 | 31 |
| Carbohydrate (g) | 83.4 | 9.0 | 77.2 | 7.5 | 80.3 | 6.0 | 165.0 | 7.7 | 156.5 | 6.2 | 161.3 | 5.2 | 184.9 | 7.5 | 174.4 | 8.1 | 180.0 | 5.5 | 220.9** | 6.2 | 194.1 | 5.3 | 205.9 | 4.1 |
| CHO (%TEI) | 47.8 | 1.0 | 45.4 | 1.8 | 46.6 | 1.1 | 53.4 | 1.0 | 53.5 | 0.9 | 53.4 | 0.7 | 52.8 | 1.0 | 55.7 | 1.0 | 54.2 | 0.7 | 55.8* | 0.5 | 53.9 | 0.6 | 54.7 | 0.4 |
| Protein (g) | 18.7 | 3.3 | 18.8 | 1.7 | 18.8 | 1.8 | 44.9 | 2.3 | 41.0 | 1.6 | 43.2 | 1.5 | 53.4** | 2.0 | 43.6 | 2.2 | 48.8 | 1.5 | 59.9 | 2.3 | 57.5 | 2.2 | 58.6 | 1.6 |
| PRO (%TEI) | 10.2 | 1.0 | 11.5 | 1.6 | 10.8 | 0.9 | 14.7 | 0.5 | 14.0 | 0.4 | 14.4 | 0.3 | 15.6** | 0.4 | 14.0 | 0.4 | 14.8 | 0.3 | 15.0 | 0.3 | 15.8 | 0.3 | 15.4 | 0.2 |
| Fat (g) | 31.6 | 1.9 | 31.7 | 1.8 | 31.7 | 1.3 | 42.5 | 1.7 | 42.3 | 2.2 | 42.4 | 1.4 | 49.4* | 2.2 | 42.0 | 2.0 | 45.9 | 1.5 | 52.0 | 1.9 | 49.4 | 1.8 | 50.5 | 1.3 |
| FAT (%TEI) | 42.9 | 1.9 | 43.9 | 1.3 | 43.4 | 1.2 | 31.8 | 0.8 | 32.4 | 0.9 | 32.0 | 0.6 | 31.3 | 0.8 | 30.2 | 0.8 | 30.8 | 0.6 | 29.1 | 0.5 | 30.1 | 0.5 | 29.6 | 0.3 |

Abbreviations: SE, standard error; %TEI, percentage of total energy intake.

Mean values were significantly different from girls of each age group based on complex sampling ANCOVA after adjusted for age: **p*<0.05, ***p*<0.01, ****p*<0.001.

Mean values were significantly different from rural children based on complex sampling ANCOVA after adjusted for age: †*p*<0.05, ††*p*<0.01, †††*p*<0.001.

Supplementary TABLE 6 Micronutrient intake by age group in boys and girls

|  | 0.5-0.9 years | | | | | | 1.0-3.9 years | | | | | | 4.0-6.9 years | | | | | | 7.0-12.9 years | | | | | |
| --- | --- | --- | --- | --- | --- | --- | --- | --- | --- | --- | --- | --- | --- | --- | --- | --- | --- | --- | --- | --- | --- | --- | --- | --- |
|  | **Boys** | | **Girls** | | **All** | | **Boys** | | **Girls** | | **All** | | **Boys** | | **Girls** | | **All** | | **Boys** | | **Girls** | | **All** | |
|  | **Mean** | **SE** | **Mean** | **SE** | **Mean** | **SE** | **Mean** | **SE** | **Mean** | **SE** | **Mean** | **SE** | **Mean** | **SE** | **Mean** | **SE** | **Mean** | **SE** | **Mean** | **SE** | **Mean** | **SE** | **Mean** | **SE** |
| All |  |  |  |  |  |  |  |  |  |  |  |  |  |  |  |  |  |  |  |  |  |  |  |  |
| Calcium (mg) | 445.5 | 39.8 | 444.8 | 40.4 | 445.1 | 28.3 | 786.0* | 30.1 | 706.0 | 24.8 | 746.0 | 19.5 | 680.1 | 27.0 | 639.9 | 24.4 | 660.0 | 18.2 | 617.8*** | 15.1 | 525.1 | 13.8 | 571.4 | 10.2 |
| Iron (mg) | 6.8 | 0.9 | 7.1 | 1.0 | 6.9 | 0.7 | 12.1* | 0.6 | 10.4 | 0.4 | 11.2 | 0.3 | 12.5* | 0.3 | 11.4 | 0.3 | 12.0 | 0.2 | 12.7** | 0.3 | 11.4 | 0.3 | 12.0 | 0.2 |
| Thiamine (mg) | 0.5 | 0.1 | 0.5 | 0.1 | 0.5 | 0.0 | 1.0* | 0.0 | 0.9 | 0.0 | 1.0 | 0.0 | 1.1** | 0.0 | 1.0 | 0.0 | 1.1 | 0.0 | 1.2*** | 0.0 | 1.0 | 0.0 | 1.1 | 0.0 |
| Riboflavin (mg) | 0.8 | 0.1 | 0.8 | 0.1 | 0.8 | 0.1 | 1.4** | 0.0 | 1.3 | 0.0 | 1.4 | 0.0 | 1.5 | 0.0 | 1.4 | 0.0 | 1.4 | 0.0 | 1.5*** | 0.0 | 1.3 | 0.0 | 1.4 | 0.0 |
| Cobalamin (µg) | 1.3 | 0.1 | 1.6 | 0.2 | 1.4 | 0.1 | 2.7 | 0.1 | 2.7 | 0.2 | 2.7 | 0.1 | 3.2 | 0.1 | 3.3 | 0.3 | 3.2 | 0.1 | 3.3 | 0.1 | 3.0 | 0.1 | 3.2 | 0.1 |
| Vitamin C (mg) | 86.9 | 10.2 | 79.3 | 7.9 | 83.1 | 6.5 | 92.4 | 5.3 | 89.3 | 4.7 | 90.8 | 3.6 | 69.9 | 4.2 | 71.6 | 4.2 | 70.7 | 2.9 | 75.3 | 4.4 | 73.9 | 3.1 | 74.6 | 2.7 |
| Vitamin A (RE) | 656.0 | 48.3 | 635.1 | 52.6 | 645.5 | 35.7 | 725.4 | 26.4 | 661.2 | 22.6 | 693.4 | 17.4 | 685.5 | 23.5 | 636.1 | 20.2 | 660.8 | 15.4 | 806.9*** | 23.9 | 678.3 | 17.9 | 742.6 | 14.9 |
| Vitamin D (µg) | 5.7 | 1.0 | 5.8 | 0.9 | 5.8 | 0.7 | 7.9 | 0.4 | 7.3 | 0.3 | 7.6 | 0.3 | 6.8 | 0.4 | 6.0 | 0.3 | 6.4 | 0.3 | 4.8* | 0.2 | 4.2 | 0.2 | 4.5 | 0.1 |
| Urban |  |  |  |  |  |  |  |  |  |  |  |  |  |  |  |  |  |  |  |  |  |  |  |  |
| Calcium (mg) | 460.4 | 45.9 | 515.7† | 52.8 | 479.6 | 34.9 | 783.7 | 34.5 | 731.5 | 29.3 | 757.9 | 22.6 | 704.7 | 32.5 | 657.1 | 29.0 | 682.4† | 22.0 | 631.1*** | 18.1 | 539.5 | 17.6 | 589.4†† | 12.8 |
| Iron (mg) | 6.8 | 0.9 | 8.6  † | 1.4 | 7.4 | 0.8 | 12.2 | 0.8 | 10.6 | 0.4 | 11.5 | 0.5 | 12.8*† | 0.4 | 11.4 | 0.4 | 12.2 | 0.3 | 13.0**† | 0.4 | 11.6 | 0.3 | 12.4†† | 0.2 |
| Thiamine (mg) | 0.5 | 0.1 | 0.6† | 0.1 | 0.6† | 0.1 | 1.0 | 0.0 | 0.9 | 0.0 | 1.0 | 0.0 | 1.2** | 0.0 | 1.0 | 0.0 | 1.1 | 0.0 | 1.3***† | 0.0 | 1.1 | 0.0 | 1.2†† | 0.0 |
| Riboflavin (mg) | 0.9 | 0.1 | 0.9†† | 0.1 | 0.9 | 0.1 | 1.4* | 0.0 | 1.3 | 0.0 | 1.4 | 0.0 | 1.5 | 0.0 | 1.4 | 0.0 | 1.5 | 0.0 | 1.5** | 0.0 | 1.3 | 0.0 | 1.4† | 0.0 |
| Cobalamin (µg) | 1.3 | 0.1 | 1.7 | 0.2 | 1.4 | 0.1 | 2.7 | 0.2 | 2.8 | 0.2 | 2.7 | 0.1 | 3.1 | 0.2 | 3.4 | 0.3 | 3.3 | 0.2 | 3.3 | 0.2 | 3.0 | 0.1 | 3.2 | 0.1 |
| Vitamin C (mg) | 95.5 | 12.7 | 94.2†† | 10.4 | 95.0†† | 9.1 | 86.9 | 5.2 | 91.1 | 5.6 | 89.0 | 3.8 | 70.9 | 5.1 | 71.8 | 4.4 | 71.3 | 3.4 | 78.1 | 4.9 | 79.4†† | 3.8 | 78.7† | 3.2 |
| Vitamin A (RE) | 698.2 | 58.6 | 729.8† | 64.0 | 709.1†† | 44.0 | 738.6 | 31.1 | 667.4 | 23.4 | 703.4 | 19.9 | 704.6 | 28.0 | 633.1 | 24.0 | 671.1 | 18.7 | 842.1***†† | 28.9 | 701.8† | 22.3 | 778.2††† | 19.0 |
| Vitamin D (µg) | 5.9 | 1.0 | 7.0 | 1.2 | 6.3 | 0.8 | 8.0 | 0.4 | 7.4 | 0.4 | 7.7 | 0.3 | 7.1* | 0.4 | 5.9 | 0.4 | 6.5 | 0.3 | 4.8 | 0.2 | 4.3 | 0.2 | 4.5 | 0.1 |
| Rural |  |  |  |  |  |  |  |  |  |  |  |  |  |  |  |  |  |  |  |  |  |  |  |  |
| Calcium (mg) | 406.3 | 77.1 | 345.3 | 46.5 | 375.8 | 46.7 | 790.6* | 58.2 | 638.2 | 44.7 | 725.5 | 39.0 | 599.1 | 42.9 | 584.6 | 40.8 | 592.3 | 29.7 | 573.3* | 24.0 | 493.3 | 21.2 | 528.6 | 16.0 |
| Iron (mg) | 6.8 | 1.9 | 4.9 | 0.9 | 5.9 | 1.1 | 11.7* | 0.7 | 9.8 | 0.6 | 10.9 | 0.5 | 11.5 | 0.5 | 11.4 | 0.6 | 11.4 | 0.4 | 11.6 | 0.5 | 10.9 | 0.5 | 11.2 | 0.3 |
| Thiamine (mg) | 0.4 | 0.1 | 0.4 | 0.1 | 0.4 | 0.1 | 1.1 | 0.1 | 0.9 | 0.1 | 1.0 | 0.1 | 1.0 | 0.1 | 1.0 | 0.1 | 1.0 | 0.1 | 1.1 | 0.1 | 1.0 | 0.1 | 1.0 | 0.0 |
| Riboflavin (mg) | 0.7 | 0.2 | 0.5 | 0.1 | 0.6 | 0.1 | 1.5* | 0.1 | 1.2 | 0.1 | 1.4 | 0.1 | 1.4 | 0.1 | 1.3 | 0.1 | 1.4 | 0.1 | 1.4 | 0.1 | 1.3 | 0.1 | 1.3 | 0.0 |
| Cobalamin (µg) | 1.4 | 0.3 | 1.3 | 0.3 | 1.4 | 0.2 | 2.8 | 0.2 | 2.5 | 0.3 | 2.7 | 0.2 | 3.3 | 0.3 | 3.1 | 0.3 | 3.2 | 0.2 | 3.3 | 0.2 | 2.9 | 0.2 | 3.1 | 0.2 |
| Vitamin C (mg) | 64.4 | 13.4 | 58.3 | 8.6 | 61.3 | 8.1 | 103.5 | 11.8 | 84.4 | 8.7 | 95.3 | 7.8 | 66.8 | 6.9 | 70.8 | 10.1 | 68.7 | 6.0 | 65.9 | 10.1 | 61.9 | 5.1 | 63.6 | 5.2 |
| Vitamin A (RE) | 545.1 | 63.2 | 502.3 | 66.5 | 523.7 | 46.8 | 699.4 | 48.2 | 644.7 | 54.7 | 676.1 | 36.5 | 622.8 | 38.7 | 646.1 | 34.9 | 633.8 | 26.2 | 689.5 | 34.8 | 626.6 | 28.9 | 654.3 | 22.2 |
| Vitamin D (µg) | 5.0 | 2.3 | 4.2 | 1.0 | 4.6 | 1.3 | 7.8 | 0.7 | 6.9 | 0.7 | 7.4 | 0.5 | 5.8 | 0.6 | 6.3 | 0.8 | 6.0 | 0.5 | 4.9* | 0.3 | 4.0 | 0.3 | 4.4 | 0.2 |

Abbreviations: SE, standard error.

Mean values were significantly different from girls of each age group based on complex sampling ANCOVA after adjusted for age: **p*<0.05, ***p*<0.01, ****p*<0.001.

Mean values were significantly different from rural children based on complex sampling ANCOVA after adjusted for age: †*p*<0.05, ††*p*<0.01, †††*p*<0.001.
